# Large Retroperitoneal Paraganglioma Associated with Germline Mutation of the Succinate Dehydrogenase Gene

**DOI:** 10.15586/jkcvhl.v8i1.129

**Published:** 2021-01-25

**Authors:** Wen Min Chen, Philip Olson, Rohith Arcot, Huy Nguyen, Faisal Quereshi, Courtney Kokenakes, Michael L. Cher

**Affiliations:** 1 Department of Urology, Wayne State University;; 2 School of Medicine, Wayne State University;; 3 Department of Pathology, Wayne State University;; 4 Department of Medical Genetics, Karmanos Cancer Center

**Keywords:** incidentaloma, paraganglioma, pheochromocytoma, retroperitoneal tumor, SDHB gene mutation

## Abstract

Paragangliomas (PGLs) are rare neural tumors that can be benign or malignant and often associated with familial syndromes. We present a case of a 23-year-old male with a large retroperitoneal PGL found incidentally during the workup of elevated liver enzymes. After surgical excision, the patient was found to have an autosomal dominant mutation in the succinate dehydrogenase B (SDHB) gene, which when compared to sporadic PGLs or other familial syndromes is associated with a higher risk of tumor recurrence, occult metastasis, and development of other cancers. The patient’s first-degree relatives were recommended to undergo screening for the genetic mutation.

## Introduction

Paragangliomas (PGLs) are highly vascular, neural crest-derived tumors that originate in the ganglia of the sympathetic and parasympathetic nervous systems or from adrenal chromaffin cells at extra-adrenal sites. When found in the adrenal gland, PGLs are better known as pheochromocytomas (PCC). These rare neural tumors can have benign or malignant potential, and while the majority occur sporadically, approximately 25% are associated with familial syndromes (1). Hereditary PGL-PCC syndromes are associated with the inheritance of mutations in the subunits A, B, C, D of the gene encoding succinate dehydrogenase (SDH). When familial inheritance is identified, prudent surveillance is needed, as increased potential arises for the development of PGLs, PCCs, and other tumors such as GIST, renal, thyroid, and breast cancer. We describe a case of a large retroperitoneal PGL found incidentally during the workup of elevated liver enzymes, in which the patient was ultimately found to have a likely pathogenic mutation in the succinate dehydrogenase B (SDHB) gene.

## Ethics Approval

Relevant approvals and exemptions were granted by institutional ethics review boards. Informed consent obtained in writing was given by the patient.

## Case Report

**Figure 1:**
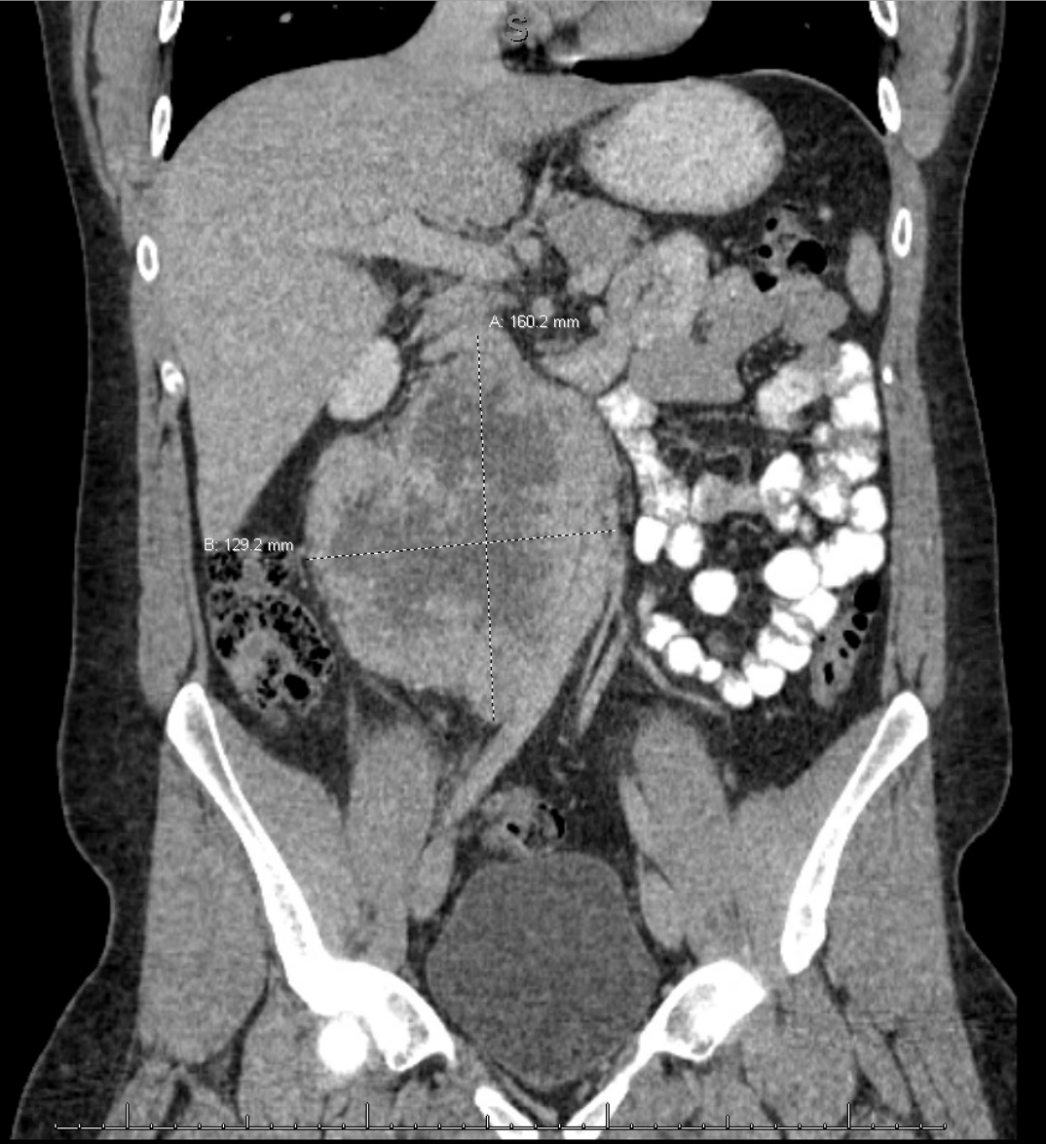
CT abdomen/pelvis with IV contrast – demonstrating 16 x 10 x 13.7 cm retroperitoneal mass encasing and displacing the IVC anteriorly, the ureter medially, and the aorta laterally. The mass extends from the renal vessels to the common iliac vessels.

A 23-year-old male in good health was found to have elevated liver enzymes during a routine physical examination. An ultrasound ordered to evaluate the liver noted the presence of a large, vascular mid-abdominal mass. Further imaging by CT confirmed a large, right retroperitoneal mass measuring 16 x 10 x 13.7 cm encasing the vena cava inferior extending from the renal veins to the bifurcation of the common iliac veins (Figure 1). Additionally, the CT noted leftward displacement of the abdominal aorta, and anterior displacement of the vena cava and lateral displacement of the right ureter. There was no evidence of metastasis to lymph nodes, bone, liver, or lung.

The CT-guided needle biopsy identified the mass as a PGL, and the patient was referred for consideration of surgery. The patient denied headaches, excessive diaphoresis, palpitations, or paroxysmal hypertension. Laboratory studies including plasma catecholamines, plasma metanephrines, 24-hour urine catecholamines and metanephrines, brain natriuretic peptide, and cortisol were normal. Although the blood work suggested no evidence of adrenergic hypersecretion, doxazosin was prescribed out of caution in preparation for surgery.

**Figure 2:**
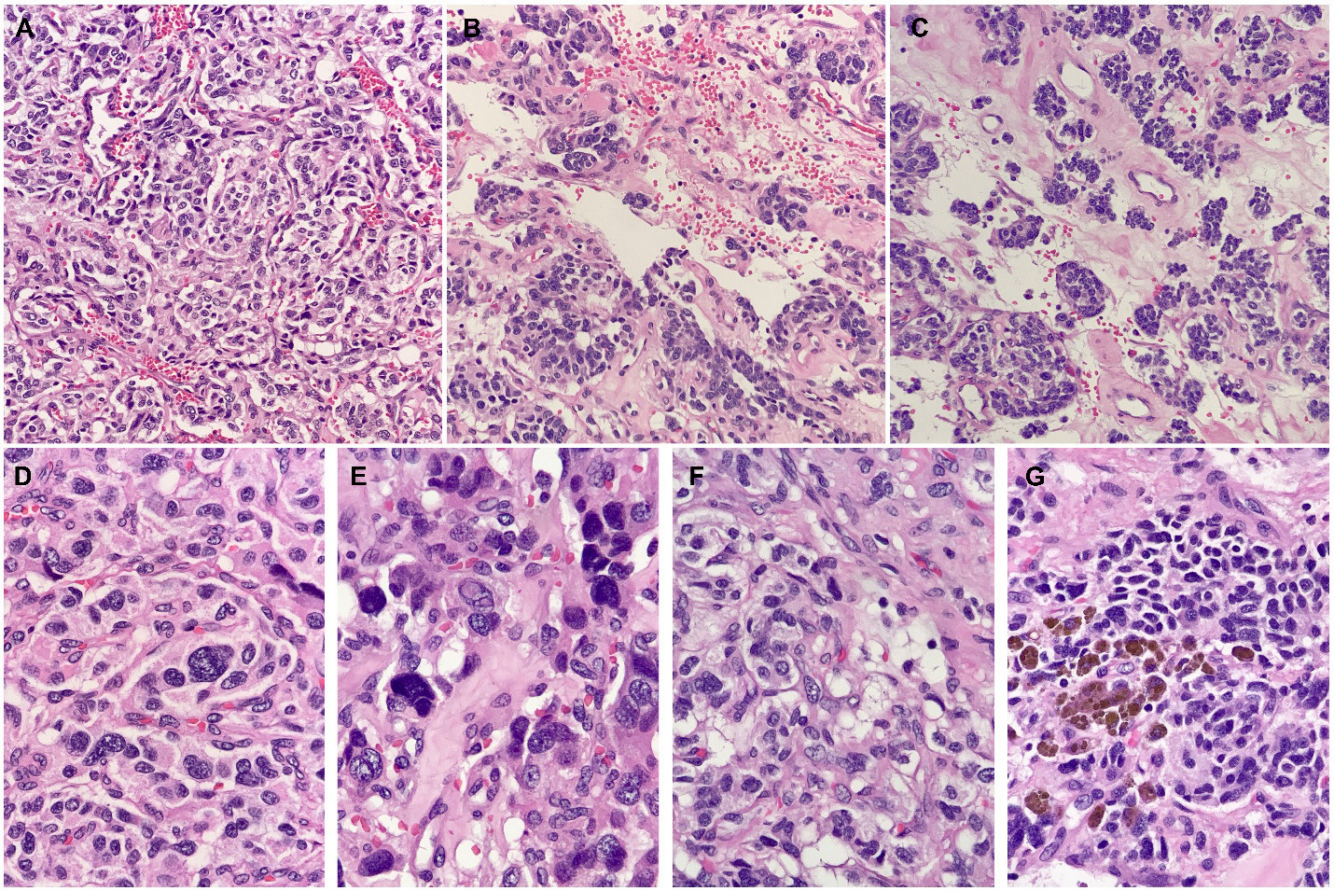
Histologic sections show: (a) The zellballen pattern (small, nested, organoid), which is characteristic of PG, features rounded nests of tumor cells within a vascularized fibrous stroma. (b) The vessels are often compressed and sinusoidal, but larger ectatic or “staghorn” vessels can also be seen. (c) Centralized areas with loose, edematous stroma can be seen within, which results in the micronodular appearance of tumor cells. Bands of collagen of variable width are also seen. (d) Tumor cells contain finely granular, eosinophilic cytoplasm and regular, centralized nuclei; some might show nucleoli. (e) Intranuclear pseudoinclusions are numerous. Nuclear enlargement and pleomorphism are also seen. These findings have no prognostic significance in isolation. (f) Clear cell change due to cytoplasmic vacuolization, sometimes quite prominent with bubbly appearance, suggests a lipoblastic differentiation. (g) Tumor cells show an accumulation of dark intracytoplasmic pigment. These pigments are often neuromelanin; however, lipofuscin and hemosiderin can also be seen (22).

**Figure 3:**
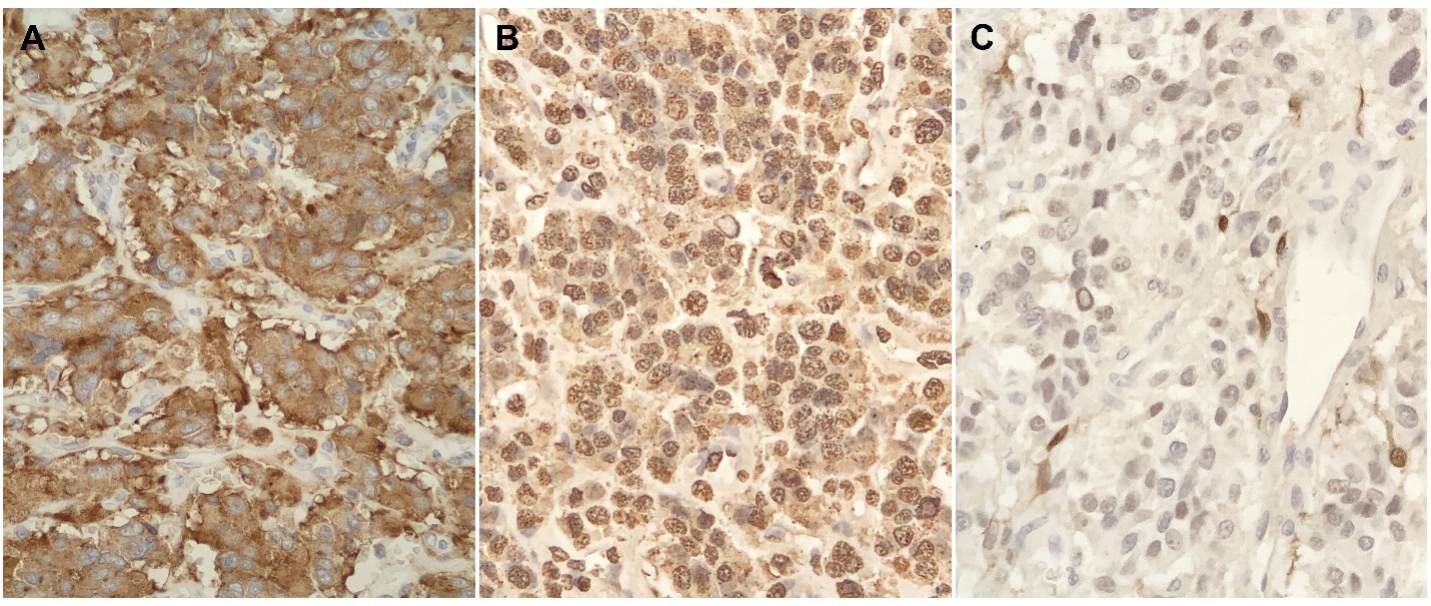
Tumor cells show diffuse expression of neuroendocrine markers, including synaptophysin (a) and chromogranin (b). S100 protein (c) highlights sustentacular cells or modified Schwann cells. These sustentacular cells surround nests of tumor cells in PG, and is characterized by thin, delicate cytoplasmic processes and nuclei.

Two months after diagnosis, the patient underwent complete excision of the mass. To minimize the risk of ureteral injury, the operation began with cystoscopic placement of a localizing right ureteral catheter. Intraoperatively, the mass was firmly adherent to the inferior vena cava (IVC) resulting in multiple vena cavotomies. The vena cava was repaired and preserved; otherwise, no viscera were injured. He was discharged home 6 days after an uncomplicated postoperative course. The pathology report confirmed the diagnosis of PGL and identified the mass to be synaptophysin and chromogranin positive (Figures 2 and 3).

**Figure 4:**
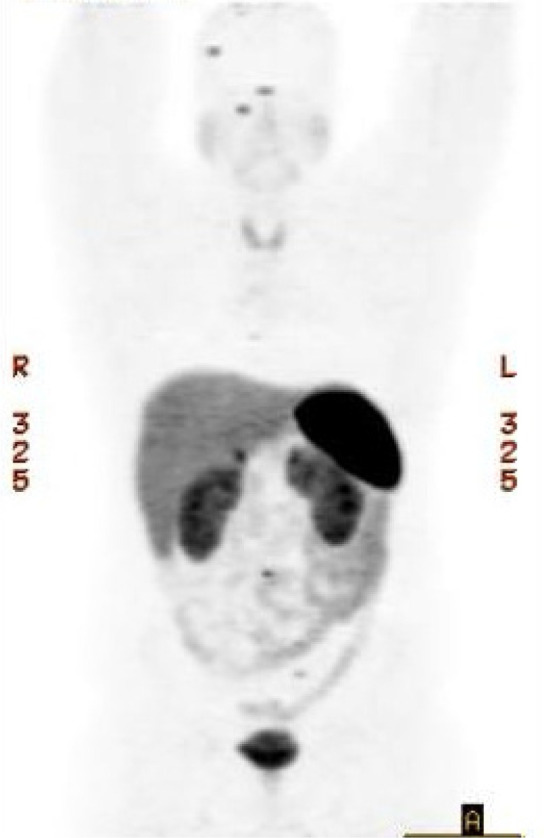
Postsurgical ^68^Ga [DOTATATE] PET scan – Demonstrating bone involvement in the right parietal temporal calvarium, right sphenoid wing, right lamina of T6, C5 vertebral body, L3 vertebral body, and left sacrum. Many of these foci do not have a definite CT correlate. No suspicious radiotracer uptake was identified within the surgical bed.

Postoperative MRI displayed interval resection of the retroperitoneal mass and no signs of metastatic disease. Due to the patient’s young age, the patient was referred for genetic counseling. Genetic testing revealed a likely pathogenic germline mutation (c.289A>T) in the SDHB gene. Thus, the patient was diagnosed with autosomal dominant PGL-PCC syndrome 4. He was informed that his syndrome confers a high risk of occult metastasis, the risk for developing reoccurrence of his PGL, as well as the development of other tumors: renal cell carcinoma, GIST, thyroid, and breast cancer. A ^68^Ga-labeled [DOTATATE] PET scan was ordered to better characterize the patient’s current disease burden. Although uptake was seen in several bone areas (Figure 4), systemic treatment will be withheld as the tempo of disease progression may be slow. The patient’s immediate family members were recommended to undergo genetic testing given the 50% risk to each first-degree relative of carrying the familial SDHB mutation.

## Discussion

Paragangliomas and their adrenal counterpart, pheochromocytomas, are rare tumors with an incidence of 0.6 cases per 100,000 persons-year. While most occur sporadically, a significant proportion can be associated with germline mutations and familial syndromes. Classically, they are associated with a constellation of symptoms attributed to the hypersecretion of metanephrines: headaches, paroxysmal hypertension, palpitations, flushing, and profuse sweating. When a PGL/PCC is suspected, the initial evaluation is completed with plasma metanephrines and CT abdomen/pelvis with IV contrast. However, in our case, the PGL was found incidentally after an abdominal ultrasound was performed for elevated liver enzymes (11). The patient’s tumor was found to be intra-abdominal and extra-adrenal which is characteristic of the more aggressive forms of sympatho-adrenal neuroendocrine tumors. PGLs can be benign tumors or malignant, which is defined by metastasis identified at the time of diagnosis since there are no pathologic or cellular markers of malignancy.

Additional imaging with positron emission tomography (PET)/CT is warranted when the tumor size is greater than >10 cm. Functional imaging with PET-CT using ^68^Ga-labeled 1,4,7,10 tetraazacyclododecane—1,4,7,10-tretraacetic acid octeotride [DOTATATE] is more effective than traditional Fluorodeoxyglucose (FDG)-PET in localizing PGLs/PCCs as well as any metastatic deposits. In our case, this was performed after the tumor was removed and showed multiple sub-centimeter boney deposits concerning metastasis (2).

Surgical resection is the mainstay of the treatment of PGLs/PCCs. In most cases, preoperative beta and alpha-adrenergic blockade is warranted. Our patient did not have elevated metanephrines but was treated preoperatively with doxazosin out of caution. As mentioned before, most PGLs are diagnosed based on clinical manifestations of catecholamine hypersecretion; however, patients with asymptomatic retroperitoneal PGLs are most commonly found incidentally on imaging or during family screening for hereditary syndromes (3). The asymptomatic nature of our patient’s mass enabled it to grow to a considerable size in the retroperitoneum.

It is estimated that up to 40% of patients who present with PGLs/PCCs carry an identifiable germline mutation. To date, at least 18 gene mutations have been identified that can result in PGLs/PCCs, of which five familial PGL syndromes have been described and named PGL-1 through PGL-5 (4, 5). The genetic defects underlying these syndromes affect the subunits of the mitochondrial enzyme succinate dehydrogenase: PGL-1 is associated with mutations in the *SDHD gene* encoding for the D subunit of succinate dehydrogenase, PGL-2 is associated with *SDHAF2*, PGL3 to *SDHC*, PGL4 to *SDHB*, and PGL5 to *SDHA* (5–9). Typically, retroperitoneal PGLs are associated with *SDHD, SDHB*, and *SDHA* mutations. Carriers of mutations in SDHB have variable penetrance (40% by age 40), often present with extra-adrenal retroperitoneal tumors, and have a high risk for metastasis at the time of presentation. The average age at diagnosis of index cases is 34, although there have been reports of index cases before the age of 10 (1). Therefore, screening may begin as early as 5–10 years of age in patients positive for *SDHB* mutation. Screening should entail a careful history and physical examination, annual measurement of blood pressure and urinary catecholamines/metanephrines, and biennial CT or MRI imaging (neck, thorax, abdomen, and pelvis) (1).

The mechanism of tumorigenesis in PGLs/PCCs is not completely understood but may be related to the hypoxia-inducible factor (HIF)/angiogenesis pathway. Succinate dehydrogenase is involved in both the electron transport chain (ETC) and the Krebs cycle. The SDH complex converts succinate to fumarate in the Krebs cycle, producing FADH_2_, which is subsequently oxidized to flavin adenine dinucleotide (FAD) by SDH in the ETC. The protein has four subunits, each having a distinct role in enzymatic function. Our patient has a mutation in the B subunit which is a hydrophilic protein that makes up a major portion of the catalytic site (4). When a mutation in the B subunit is present, the conversion of succinate to fumarate is disrupted leading to a buildup of succinate in the cell. The excess succinate diffuses from the mitochondria to the cytoplasm and inhibits prolyl hydroxylase, which stabilizes the HIF (4). HIF is a transcription factor that modulates the expression of genes responsible for pro-angiogenic factors, cell proliferation, glycolytic metabolism, and proteins involved in metastasis and tumor cell dedifferentiation (10).

The management of patients found to have SDHB gene mutations must be tailored to the individual since presentation can be varied: age of presentation, location of the primary tumor, metastases at diagnosis, benign PGLs, and identification of concomitant non-PGL tumors. Under the direction of a multi-disciplinary team, both medical and surgical options should be utilized (11). After resection, close surveillance is particularly warranted in patients with PGL4. When compared to other PGL syndromes, PGL4 has a higher rate of metastasis at diagnosis, risk of recurrence, and risk of developing other primary tumors due to SDHB’s role as a tumor suppressor (11, 12). Associations have been found between PGL4 and the development of renal cell carcinoma (14%), gastrointestinal tumors (GIST) (2%), and head and neck PGLs (20–30%) (5).

After resection of a PGL/PCC long-term surveillance should be conducted based on the SDH subunit-specific mutation identified. For carriers of SDH(B) mutation, there is much debate regarding the frequency and modality of surveillance, since there is variable penetrance and limited long-term follow-up data in the literature. Finally, missing a significant lesion could result in metastatic disease with poor prognosis. It is recommended that at the time of diagnosis a metastatic workup with functional imaging be conducted (13, 14). If no metastatic disease is found, lifelong surveillance is recommended, preferably with MRI to avoid cumulative radiation exposure. The frequency of imaging is still highly debated, however, a systematic review of surveillance protocols for asymptomatic SDH mutation carriers found that abdominal MRI every 18 months with MRI of the neck, thorax and pelvis every 3 years is unlikely to miss a significant lesion (13, 14).

In the event metastatic disease is found, radiotherapy, chemotherapy, and immunotherapy are viable treatment options. Radiotherapy, in the form of peptide receptor radionuclide therapy (PRRT), uses drugs or substances tagged to radionuclides that bind to specific peptide receptors that are overexpressed on cancers cells causing targeted cell death with minimal side-effects (15). High-specific-activity I-131-MIBG (HSA-I-131-MIBG) is a norepinephrine analog that is taken up by the noradrenaline transporter expressed in neuroendocrine cells and is the standard of care for patients with MIBG-avid tumors (16). Currently, HSA-I-131-MIBG is the only FDA approved therapy in the US for metastatic PCC and PGLs, and is the focus of promising future research regarding efficacy enhancement as only around a third of patients can see a clinical benefit (16). Astatine-211-labeled MIBG (At-211) is another type of PRRT that uses alpha particles as opposed to the standard beta particles used in radionuclide therapy allowing for a higher linear energy transfer and cytotoxicity (15). At-211 has yet to undergo clinical utility studies but has significant therapeutic potential as a novel PRRT agent. Additionally, chemotherapy using cyclophosphamide, vincristine, and dacarbazine (CVD) remains an option for metastatic neuroendocrine tumors. A metaanalysis performed by Niemeijer et al. (17) found that CVD chemotherapy was able to produce partial response concerning tumor volume in 37% of patients and partial response concerning catecholamine excess in 40% of patients. Furthermore, the complete response for tumor volume and catecholamine excess was found to be 4 and 14%, respectively. The alkylating agent temozolomide has also shown promise in treating metastatic neuroendocrine tumors, with one study reporting a 70% reduction in tumor size in a SDHB-mutation-positive patient (18). Side effects of these chemotherapy regimens including myelosuppression, peripheral neuropathy, and gastrointestinal toxicity can lead to high rates of treatment discontinuation. This can be exacerbated if the cancer is nonresponsive to CVD chemotherapy initially, leading to a significantly low number of cycles tolerated by these patients. Overall, the systemic side effects in conjunction with the inconsistent response rate among patients prevent chemotherapy from being used as a first-line agent. Usually, it is reserved for patients with a high tumor burden or a large number of bone metastases (19). Sunitinib, a receptor tyrosine kinase inhibitor, which targets receptors for a vascular endothelial growth factor (VEGF), has also been studied as a systemic treatment option for metastatic disease. Favier et al. (19) found that increased VEGF expression reflected an overrepresentation of SDHB-related tumors. While not yet approved for PCC/PGL, a preliminary study of sunitinib has shown significant therapeutic benefits in patients including a reduction in tumor size, decreased FDG-PET/CT uptake, disease stabilization, and improvements in hypertension (20).

Since mutations in the *SDHB* gene are inherited in an autosomal dominant manner, genetic testing and counseling are an integral part of multi-disciplinary care. Following surgical resection, the patient met with a genetic counselor to begin this process. The patient was born to a mother and father of Lebanese descent without consanguinity. Family history was significant for prostate cancer in father and paternal uncle in their 50s, and breast cancer in maternal aunt in her 40s. Notably, the patient did report a half-brother on his father’s side who had surgical resection of an abdominal mass, but pathology was unknown and the brother is no longer in contact with the family. The patient was informed that all his first-degree relatives should be screened and undergo surveillance if they test positive for the mutation (21). To date, no family members have undergone genetic testing despite being encouraged to do so.

Following the initial PET scan showing multiple sub-centimeter bone metastasis, the patient received two repeat PET scans 2 months apart, both revealing stable disease. Treatment is not being considered at this time as the patient’s disease course appears to be indolent, and he remains asymptomatic. As there are no CT correlates of the metastasis, a bone biopsy would be challenging and may not be able to confirm the diagnosis. The patient will follow up with a repeat PET scan in 4 to 6 months.

## Conclusions

We present a case of a large, asymptomatic retroperitoneal tumor identified by abdominal imaging with ultrasound. Ultimately, the patient had successful surgical resection of the mass which was confirmed to be a PGL. Genetic testing revealed a mutation in the SDHB subunit consistent with a diagnosis of autosomal dominant PGL4 syndrome. As a result, the patient is at a high risk of recurrence or the development of other primary tumors. Additionally, the patient’s first-degree relatives should be screened and undergo surveillance if they test positive for the mutation. Unfortunately, following resection of his primary tumor ^68^Ga [DOTATATE] PET scan identified multiple small boney lesions concerning metastasis. What remains unknown is the oncologic potential of these boney deposits and an indolent course lies ahead.
